# Comparative analysis of proximate compositions, mineral and functional chemical groups of 15 different seaweed species

**DOI:** 10.1038/s41598-022-23609-8

**Published:** 2022-11-15

**Authors:** Amal D. Premarathna, Rando Tuvikene, P. H. P. Fernando, Ranjith Adhikari, M. C. N. Perera, T. H. Ranahewa, Md Musa Howlader, Phurpa Wangchuk, Anura P. Jayasooriya, R. P. V. J. Rajapakse

**Affiliations:** 1 School of Natural Sciences and Health, Tallinn University, Narva mnt 29, 10120 Tallinn, Estonia; 2 Department of Veterinary Pathobiology, Faculty of Veterinary Medicine and Animal Science, University of Peradeniya, Peradeniya, Sri Lanka; 3 Department of Biochemistry, Faculty of Medicine, University of Peradeniya, Peradeniya, Sri Lanka; 4 National Serpentarium, South Asian Clinical Toxicology Research Collaboration, Faculty of Medicine, University of Peradeniya, Peradeniya, Sri Lanka; 5 Centre for Molecular Therapeutics, Australian Institute of Tropical Health and Medicine, James Cook University, Smithfield, QLD 4878 Australia; 6 Department of Basic Veterinary Sciences, Faculty of Veterinary Medicine and Animal Science, University of Peradeniya, Peradeniya, Sri Lanka

**Keywords:** Biochemistry, Lipids

## Abstract

Seaweed is a popular edible source and is associated with many foods and pharmaceutical industries around the world. The current research aims to provide information on the chemical composition of 15 seaweed species, consisted of Chlorophyta, Ochrophyta/Phaeophyceae, and Rhodophyta macroalgae, collected from coastal areas of Sri Lanka. Seaweed samples were subjected to the analysis of lipids, proteins, ash and macro, micro, trace and ultra-trace elements. The highest protein content was recorded in the brown algae. Maximum dietary fiber and ash contents were recorded from green algae. The highest predominant fatty acids were observed from green seaweeds (*Caulerpa* *racemosa*); however, linoleic acid (C18:2n6) is the dominant fatty acid of all macroalgae. Mineral contents were highest in the red macroalga; however, copper, zinc and magnesium were also comparatively higher in green alga *Ulva* *lactuca*. In conclusion, 15 seaweed species belonging to the three different classes of seaweeds are investigated in details to obtain their biochemical, mineral and fatty acid compositions for the synthesis of novel therapeutic agents. In order to explore biorefinery processes for these seaweeds, as well as how they can potentially be cultivated, more extensive studies are required. Studying and determining the nutritional values of seaweeds will be beneficial with the potentials for future industrial uses and research.

## Introduction

Seaweeds are classified into three taxonomic groups Chlorophyta (green algae), Ochrophyta (Phaeophyceae; brown algae) and Rhodophyta (red algae), mainly based on their pigmentation and morphological features^1^. Conventionally, seaweeds have been used as medications, valuable food commodities, fertilizer supplements and animal feeds^2^.

They have been used as edible commodities due to their ability to reduce the risks of many non-communicable diseases^3^. Further, studies by Honkanen^4^ demonstrated enormous health benefits of fresh or dried seaweeds. Seaweeds have been known to contain bioactive compounds^5^ that are derived from sulfated polysaccharides, polyphenols, carotenoids, proteins and lipids^6^. Seaweeds have demonstrated anti-inflammatory^7^, wound healing^8,9^, anti-cancer^10^, anti-diabetic activities, and anti-degenerative activities^6^. Isolated compounds of seaweeds have been subjected to clinical trials to investigate their potential drug abilities in the field of oncology^11^. Green, brown and red algae are known for their antiviral, anthelminthic, antibacterial and antifungal activities^12^. Moreover, pharmaceutical preparations of seaweeds have been introduced to the market as a result of recent investigations^13^. Polyunsaturated fatty acids (PUFAs) regulate a wide range of functions, including inflammatory responses^14^, blood pressure, blood clotting, brain development^15^, and nervous system regulation^16^. It has been observed that fish oil is the major source of n-3 and n-6 long-chain PUFAs, such as arachidonic acid, EPA, and DHA. These long-chain PUFAs are not originally derived from fish, but rather from marine algae and phytoplankton that are primarily their food source^17^.

Seaweed is a rich source of essential mineral and trace elements^18^. Brown algae can be used as a food reserve to fulfil the daily intake of some minerals (Na, K, Ca, Mg) and trace elements (Fe, Zn, Mn, Cu)^19^. Marine algae are good potential sources of polysaccharides and dietary fibres, lipids, proteins, essential amino acids and vitamins A, B, C, and E^20^. Seaweeds also contain many essential fatty acids. Red and brown algae, for instance, are particularly rich in omega-3 and -6 fatty acids, EPA and α-linolenic acid, and linoleic acid, along with relatively high levels of oleic and palmitic acids^21^. In addition, chemical composition of some seaweed species make them sources of herbicides, and chemicals useful in various industrial applications^22^.

There is a knowledge gap on nutritional properties and chemical composition of seaweeds originating from the coastal waters of Sri Lanka. Therefore, this study was aimed to gather such information to favour potential development of seaweed farming in the area Furthermore, our previous study was able to establish data on the diversity and distribution of various seaweed species in Sri Lanka^23^. In addition, few numbers of surveys have recently been conducted to identify and map their bathometric division in the sea of Sri Lanka. The dominant seaweeds found in Sri Lanka belong to Phaeophyceae (*Sargassum* spp.*,* *Padina* spp.*,* *Turbinaria* sp.*,* and *Stoechospermum* spp.), Chlorophyta (*Caulerpa* spp*.,* *Chaetomorpha* spp*.,* *Ulva* spp*.* and *Halimeda* spp.) and Rhodophyta (*Gracilaria* spp*.,* *Acanthophora* spp*.,* *Gelidiopsis* spp., and *Jania* spp.)^23^. Given the tremendous potential for the extensive culture of native seaweeds along Sri Lanka's coastal waters, a first and crucial step would be to determine the chemical composition of them in order to ascertain their potential usage as a food and feed sources as well as their potential industrial applications based on agar, alginate, and carrageenans. Another study conducted in Sri Lanka using twenty-three seaweed species demonstrated a high rate of cell proliferation, migration, toxicity and wound healing properties when studied in the in-vitro and in-vivo experimental models^23^. According to the authors' knowledge, no information regarding the chemical composition of seaweeds from Sri Lanka has been published. Based on these preliminary findings, we investigated the functional chemical groups, proximate composition, and fatty acid and mineral content of representative green, brown, and red seaweeds collected from the Sri Lankan shore and we emphasized on their prospective therapeutic uses.

## Materials and method

### Samples collection and preparation

**Figure 1:**
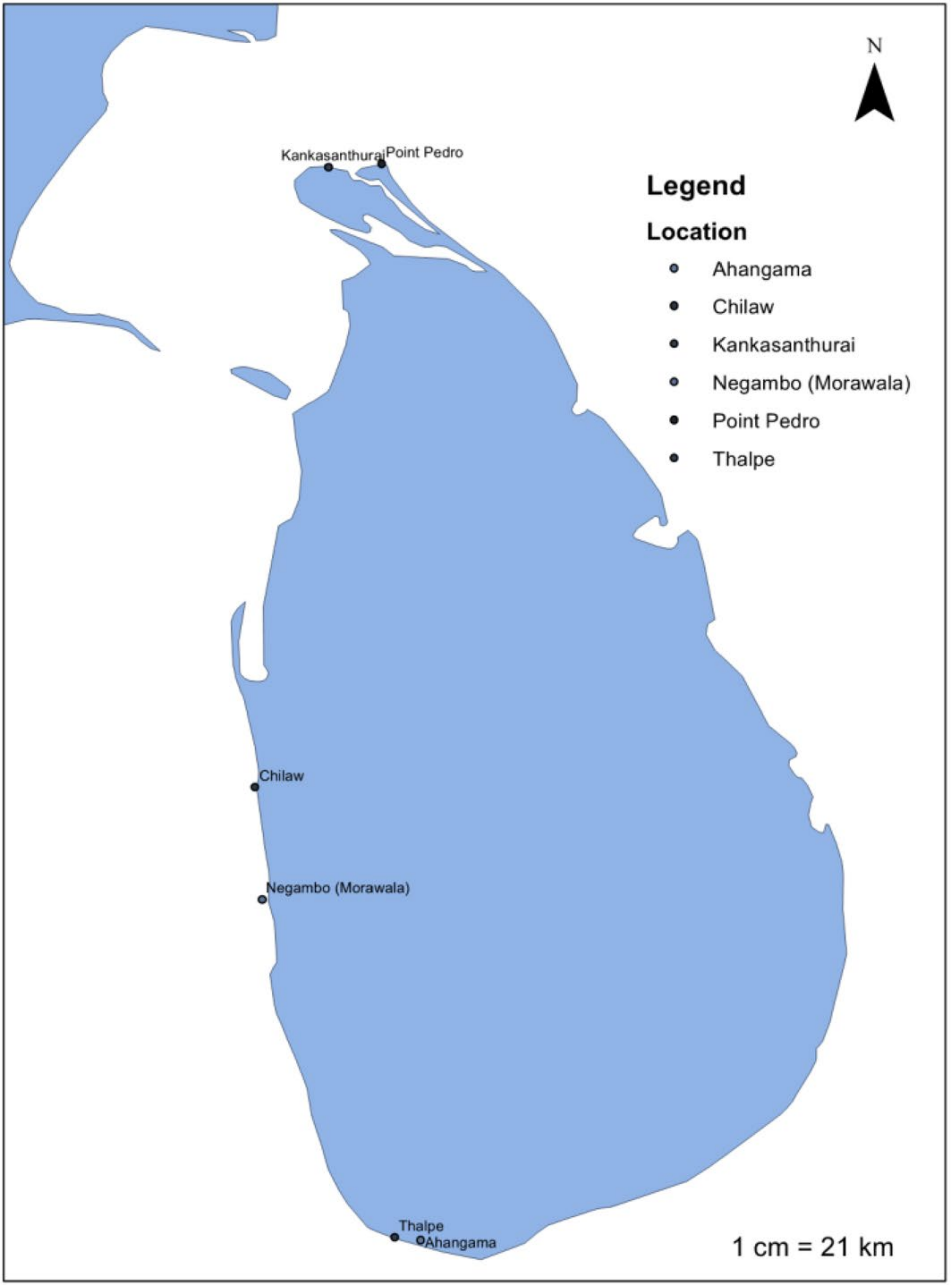
Map of seaweed samples collection location. (01) Ahangama (N 05°58.006' E 080°22.482'), (02). Thalpe (N 05°59.792' E 080°16.898'), (03) Chilaw (N 07°36.220' E 079°47.120'), (04) Negombo (N 07°12.170' E 079°48.570'), (05) Kankasanthurai (N 09°48.592' E 080°02.546'), (06) Point Pedro (N 09°49.501' E 080°15.119') (ArcGIS for Desktop 10.3.1 version was used to create the map—https://www.esri.com/en-us/arcgis/products/arcgis-desktop/overview).

Seaweed species samples, belonging to Ochrophyta/Phaeophyceae (Brown), Chlorophyta (Green) and Rhodophyta (Red), were collected from Northern, Southern and North-western coastal sites of Sri Lanka (Table 1, Fig. 1). The fresh seaweed samples were then washed thoroughly with tap water to remove all sand particles and epiphytes. Then these seaweed samples were air dried at 40 °C until consistent weights were obtained. Next, each sample was ground using an electrical grinder to prepare < 0.5 mm particle size powder. After that the prepared powder samples were freeze-dried for four days and later, they were milled till they become fine powder (using water-cooled mill) and samples were stored at −20 °C until further use.

**Table 1:** Name of seaweed species, collected location and voucher numbers.

Type and voucher number	Species name	Location	GPS point
**Chlorophyta**			
G1	*Ulva* *lactuca*	Thalpe	N 05°59.792' E 080°16.898'
G2	*Caulerpa* *racemose*	Ahangama	N 05°58.006' E 080°22.482'
G3	*Halimeda* *opuntia*	Kankasanthurai	N 09°48.592' E 080°02.546'
G4	*Caulerpa* *racemose*	Point Pedro	N 09°49.501' E 080°15.119'
G5	*Caulerpa* *sertularioides*	Kankasanthurai	N 09°48.592' E 080°02.546'
G6	*Ulva* *lactuca*	Negombo	N 07°12.170' E 079°48.570'
G7	*Chaetomorpha* *antennina*	Chilaw	N 07°36.220' E 079°47.120'
G8	*Chaetomorpha* *crassa*	Chilaw	N 07°36.220' E 079°47.120'
**Phaeophyta**			
B1	*Padina* *antillarum*	Ahangama	N 05°58.006' E 080°22.482'
B2	*Sargassum* *ilicifolium*	Thalpe	N 05°59.792' E 080°16.898'
B3	*Sargassum* *polycystum*	Point Pedro	N 09°49.501' E 080°15.119'
B4	*Turbinaria* *ornate*	Kankasanthurai	N 09°48.592' E 080°02.546'
B5	*Stoechospermum* *polypodioides*	Point Pedro	N 09°49.401' E 080°14.593'
B6	*Sargassum* *ilicifolium*	Kankasanthurai	N 09°48.592' E 080°02.546'
B7	*Sargassum* *ilicifolium*	Negombo	N 07°12.170' E 079°48.570'
B8	*Padina* *antillarum*	Negombo	N 07°12.170' E 079°48.570'
B9	*Sargassum* *ilicifolium*	Ahangama	N 05°58.006' E 080°22.482'
**Rhodophyta**			
R1	*Gracilaria* *corticate*	Ahangama	N 05°58.006' E 080°22.482'
R2	*Gracilaria* *corticate*	Negombo	N 07°12.170' E 079°48.570'
R3	*Acanthophora* *spicifera*	Negombo	N 07°12.170' E 079°48.570'
R4	*Gelidiopsis* *variabilis*	Chilaw	N 07°36.220' E 079°47.120'
R5	*Gracilaria* *corticate*	Chilaw	N 07°36.220' E 079°47.120'
R6	*Jania* *adhaerens*	Chilaw	N 07°36.220' E 079°47.120'

### Analytical methods

Experiments were carried out to measure the contents of moisture, dry matter, ash, dietary fiber, lipid and protein present in the prepared samples. Each chemical assay experiment for an individual seaweed sample was performed in triplicates.

#### Analysis of moisture content

The seaweed samples were dried at 40 °C in air dryer machine until persistent weight was obtained. The moisture content was determined by oven (Victor, England) drying method at 105 °C.

#### Analysis of ash content

Dried samples that were prepared for measuring the moisture content was used for determining the total ash content. The method described by Pomeranz and Meloan^24^ was applied to quantify the crude ash content of seaweed samples. Consequently, the samples were burnt according to the protocol and ashes were kept in a muffle furnace (Gallenkamp, England) at 550 °C for 6 h until a constant weight is gained.

#### Analysis of protein content

We used the method described by Smith et al.^25^ to measure protein content of the seaweed samples. the standards were prepared using Bovine Serum Albumin (BSA) in the following steps: 1 mL of BSA stock (2 mg mL^−1^ in water) was prepared and serial dilutions (five–eight) were made with concentrations ranging from 20 to 2000 μg mL^−1^. Bicinchoninic Acid (BCA) working reagent (WR) was prepared by calculating the total volume of WR needed. To make WR, 50 parts of BCA reagent A was mixed with 1 part of BCA reagent B (50:1, reagent A:B) (the mixture appeared bright green). It was necessary to prepare a sample to WR ratio of 1:20 by using 200 μL of WR reagent. The wells were filled with 10 μL of each standard or protein sample replicate. For standard curves and protein samples, buffers used for preparation of protein samples were used as blank solutions. Then, 200 μL of WR was added to each microplate well (96 well plate). Consequently, tubes were covered and incubated at 37 °C for 30 min. After incubation, it was kept at room temperature for 10 min before the measurements were taken. As the final step, absorbance was measured using an ELISA reader (Muitiskan Ex, German) in 562 nm wavelength.

#### Analysis of lipid content

In order to quantify the lipid content of the seaweed samples, a chloroform:methanol (2:1, v/v) extraction process was performed according to the method described by Folch et al.^26^. Therefore, 1 g of dried powder sample was placed in a test tube, followed by the addition of a mixture of 6 ml of chloroform and 3 mL of methanol, which was then vortexed for 90 s and 30 s intervals. The mixture was incubated at 40 °C in an ultrasonic water bath (Branson 2510, Danbury, USA) for 30 min. Then, 3.75 mL of a 2% NaCl solution and 3.75 mL of chloroform (1:1) were added. This sample was shaken vigorously in a shaker (Kika-labortechnik, Germany) for few minutes. Once the sample had been shaken vigorously, it was centrifuged (Beckman Avanti, UK) at 1700*g* for 20 min, and the chloroform layer at the bottom was removed with the pasteur pipette into an extraction tube. The mixture of chloroform and methanol was then allowed to evaporate. The residue inside the tube was measured for their weights.

##### Preparation of fatty acid methyl esters

The fatty acid composition was determined as the methyl esters of fatty acids by gas chromatography (GC). Free fatty acids were obtained by the fatty acids methyl esters (FAMEs) extraction method described by Levy et al.^27^ and the method modified by Premarathna et al.^28^. Firstly, approximately 100 mg of seaweed oil/extract was weighed into a separate glass tube and added 3 mL of 5% H_2_SO_4_ in methanol. Then the mixture was heated at 50 °C for 1 h in an oven and it was gently shaken for 30 s after every 15 min intervals. Once the one-hour cycle was completed, the sample was placed on a cold water/crushed ice layer. The distilled water (2 mL) and hexane (3 mL) was added to the tube, then the sample was shaken thoroughly and allowed to separate into two layers. Thereafter, 2 mL of the upper layer was drawn into a clean glass tube and was allowed to evaporate using N_2_ gas. As the final step in this procedure, 500 μL of hexane was added to the tube. The tube with the solution was labelled and stored in the freezer (−20 ºC) until subjected to GC analysis.

##### Analysis of fatty acid composition

A Thermo Scientific TRACE 1300 Gas chromatography equipped with a Flame Ionization Detector (FID) and a fused silica capillary column omega (30 m × 0.25 mm internal diameter and 0.20 μm film thickness) with temperature limits in the range of 40 to 240 °C was used to analyse the FAME samples. Helium was used as carrier gas with a flow rate of 3 °C m/s. The temperature was set at 250–280 °C for both injector and detector (Carrier mode, Flow control, Spit mode, Splitless, column flow; 1.000 mL/min, purge flow; 5.000 mL/min, Spit flow; 10.0 mL/min). Injection was performed in splitless mode with a volume of 1 μL. The values were always averaged over at least three injections of each duplicate extract. The individual fatty acids' concentrations were calculated and expressed as mass percentages of total identified fatty acids. Peaks of the gas chromatogram are proportional to fatty acid quantities and total fatty acids. Finally, to identify and quantify fatty acids, we used FAME Mix (PUFA-2Animal source, Catalog No: 47015-U) as standards.

#### Determination of macro, trace and ultra-trace elements

Total of seventeen minerals (Co, Mg, Cr, Ni, Cd, Cu, Mn, Fe, Zn, Pb, Mo, Li, Se, Sr, Na, K and Ca) were determined in the seaweed samples using iCE 3000 series atomic absorption spectrometers (AAS), graphite furnace technique. Exactly 0.1 g of dry ash sample was weighed into a glass tube, and 10 mL concentrated nitric acid (HNO_3_) was added. The mixture was allowed to stand for few hours until the content become colourless. The digested material was then filtered through Whatman (No. 40) filter paper. The collected 2 mL filtrate was serially diluted with deionized distilled water to reach 5 mL and the minerals were detected by spectrometry (iCETM 3000, Thermo Scientific).

#### FTIR spectra acquisition

The FTIR spectra of seaweed materials were recorded using the iS50 (Nicolet) Fourier infrared spectrophotometer (Thermo Fisher Scientific, Waltham, MA, USA) after milling dried samples by cryomilling. The spectra were scanned at room temperature in absorption mode at the wavelength of 400–4000 cm^−1^, with the Omnic software version 9.2.

### Statistical analysis

Statistical analyses was conducted using GraphPad Prism 6 software and Data management and descriptive statistics were done using Microsoft Excel (Microsoft, Redmond WA, USA). Multiple groups were compared using either one-way or two-way analysis of variance depending on the number of variables, followed by Tukey’s multiple comparison test. Statistical significance was achieved when P < 0.05. All data were presented as mean ± standard error of mean (SEM).

## Results

A comprehensive analysis of 15 different Sri Lankan seaweed species (Table 1), belonging to Ochrophyta/Phaeophyceae, Chlorophyta, and Rhodophyta, were undertaken to determine their potential for nutritional (feed, etc.) and medicinal purposes. The mean dry matter, ash, total protein, lipid and total dietary fiber percentage of seaweeds are shown in Table 2. In the present study, the maximum protein content was recorded in the brown algae while, the minimum protein content was reported from green algae (Table 3). In this study, protein content varied between 15.64 ± 2.11% in green algae, 26.69 ± 2.21% in red algae, and 24.13 ± 6.30% in brown algae. Protein content ranged from 06.89 to 62.04%, where brown seaweeds contained more proteins, followed by the red and green seaweeds. Indeed, the protein content of brown algae species, *Sargassum* *ilicifolium* (B2) 28.02 ± 0.68%, and *S.* *ilicifolium* (B9) 30.31 ± 0.58 showed variable amount of nutritional properties according to different oceanographic terrain in Sri Lanka”. High proportions of total dietary fiber were reported from all the 15 seaweed species, with the highest content variation observed in green alga, *Caulerpa* *racemosa*. The ash content was higher in green algae than in red algae.

**Table 2:** Proximate composition values and the respective seaweed species (mean ± SE).

Seaweed sample no.	Seaweeds species	Moisture	Dry matter	Ash	Lipid	Protein	Total dietary fiber
**Chlorophyta**							
G1	*Ulva* *lactuca*	89.67 ± 0.64	9.51 ± 0.06	01.41 ± 0.04	1.37 ± 0.04	06.89 ± 0.23	81.59 ± 0.40
G2	*Caulerpa* *racemosa*	98.08 ± 0.35	2.25 ± 0.03	05.41 ± 0.35	3.73 ± 0.19	08.77 ± 0.34	81.70 ± 0.91
G3	*Halimeda* *opuntin*	98.92 ± 0.34	0.73 ± 0.02	47.36 ± 0.57	0.03 ± 0.001	16.72 ± 0.40	35.72 ± 0.55
G4	*Caulerpa* *racemosa*	95.19 ± 0.25	5.14 ± 0.06	20.72 ± 0.67	6.70 ± 0.11	23.78 ± 0.28	44.63 ± 0.61
G5	*Caulerpa* *sertularioides*	97.06 ± 0.40	3.40 ± 0.12	24.57 ± 0.50	1.63 ± 0.21	22.05 ± 0.55	48.46 ± 0.41
G6	*Ulva* *lactuca*	96.64 ± 0.02	3.37 ± 0.02	10.25 ± 0.17	1.48 ± 0.05	16.30 ± 0.80	68.63 ± 0.41
G7	*Chaetomorpha* *antennina*	94.08 ± 0.02	5.86 ± 0.06	42.29 ± 0.69	1.58 ± 0.17	18.14 ± 0.70	32.62 ± 0.69
G8	*Chaetomorpha* *crassa*	96.06 ± 0.53	3.03 ± 0.02	32.50 ± 1.04	1.27 ± 0.04	12.49 ± 0.18	52.70 ± 0.67
**Phaeophyta**							
B1	*Padina* *antillarum*	96.97 ± 0.34	3.25 ± 0.06	05.26 ± 0.16	4.25 ± 0.10	19.66 ± 0.30	67.59 ± 0.48
B2	*Sargassum* *ilicifolium*	95.92 ± 0.37	4.34 ± 0.05	13.15 ± 0.41	4.45 ± 0.12	28.02 ± 0.68	51.46 ± 0.53
B3	*Sargassum* *polycystem*	92.58 ± 0.32	7.58 ± 0.11	18.48 ± 0.21	4.50 ± 0.21	16.15 ± 0.33	54.49 ± 0.95
B4	*Turbinaria* *ornate*	95.74 ± 0.41	3.79 ± 0.07	08.58 ± 0.20	3.33 ± 0.08	23.54 ± 0.53	62.04 ± 0.58
B5	*Stoechospermum* *polypodioides*	92.21 ± 0.01	7.72 ± 0.03	10.21 ± 0.47	5.63 ± 0.16	08.02 ± 0.26	68.63 ± 0.61
B6	*Sargassum* *ilicifolium*	96.17 ± 0.59	3.76 ± 0.06	05.33 ± 0.28	2.51 ± 0.04	43.87 ± 0.37	45.32 ± 0.42
B7	*Sargassum* *ilicifolium*	94.40 ± 0.64	6.18 ± 0.02	11.06 ± 0.96	1.54 ± 0.04	22.89 ± 0.33	58.92 ± 0.95
B8	*Padina* *antillarum*	96.04 ± 0.58	5.07 ± 0.04	41.83 ± 0.26	2.35 ± 0.03	24.83 ± 0.40	26.64 ± 0.49
B9	*Sargasum* *ilicifolium*	96.07 ± 0.54	4.45 ± 0.05	08.43 ± 0.13	3.30 ± 0.09	30.31 ± 0.58	55.29 ± 0.59
**Rhodophyta**							
R1	*Gracilaria* *corticata*	95.30 ± 1.22	4.10 ± 0.04	08.17 ± 0.49	1.80 ± 0.04	26.08 ± 0.64	61.59 ± 0.72
R2	*Gracilaria* *corticata*	94.82 ± 0.89	5.51 ± 0.02	21.98 ± 0.23	2.34 ± 0.17	16.09 ± 0.30	54.65 ± 0.58
R3	*Acanthophora* *spicifera*	95.02 ± 0.54	4.87 ± 0.03	13.32 ± 0.01	3.49 ± 0.13	28.89 ± 0.35	48.83 ± 0.91
R4	*Gelidiopsis* *variabilis*	94.52 ± 0.72	5.09 ± 0.05	21.64 ± 0.03	2.13 ± 0.04	30.90 ± 0.23	40.42 ± 0.60
R5	*Gracilaria* *corticata*	96.32 ± 0.02	3.57 ± 0.05	07.15 ± 0.01	1.66 ± 0.18	28.70 ± 0.46	59.15 ± 0.76
R6	*Jania* *adhaereus*	92.96 ± 0.27	7.21 ± 0.02	05.01 ± 0.01	1.52 ± 0.08	29.47 ± 0.15	56.81 ± 0.38

**Table 3:** Proximate composition values and the respective seaweed groups (mean ± SE).

Chemical composition %	Green seaweed	Brown seaweed	Red seaweed
Moisture	95.71 ± 1.02	95.12 ± 0.56^ac†^	94.82 ± 0.45
Dry matter	04.16 ± 0.95	05.13 ± 0.55	05.06 ± 0.51
Ash	23.06 ± 5.98^bc*^	13.59 ± 3.78	12.88 ± 3.04
Lipid	02.22 ± 0.73	3.543 ± 0.43	02.15 ± 0.29
Protein	15.64 ± 2.11^bc*^	24.13 ± 6.30	26.69 ± 2.21
Total dietary fiber	56.13 ± 6.05	54.48 ± 6.58	53.57 ± 3.18

Data are expressed as values: mean ± SE of three replicates and analysed by one-way analysis of variance. *a* = when compared with green algae group, *b* = when compared with brown algae group, *c* = when compared with red algae group, (*) indicates statistically significant difference from respective group using ANOVA, followed by Tukey comparisons test (p > 0.05). (†) indicates statistically no significant difference from respective group using ANOVA, followed by Tukey comparisons test (p > 0.05).

In this study, lipids were recorded to be the least available component from majority of the seaweed species. The highest and lowest lipid contents were recorded from the species of Chlorophyta: *Caulerpa* *racemose* and *Halimeda* *opuntia,* respectively (Table 2). A total of 17 identifiable fatty acids were recorded in this study (Table 4). Palmitic acid (C16:0) was the dominant saturated fatty acid that was detected in this study. While palmitoleic acid (C16:1n7) was the main monounsaturated fatty acid, linoleic acid (C18:2n6) and homo-γ-linolenic (C20:3n6) were the major polyunsaturated fatty acid (PUFA) present in Rhodophyta species. Among the PUFA, docosahexaenoic acid (DHA) (C22:6n3) was present in lowest concentration (Table 5).

**Table 4:** The percentage as a mean value of fatty acid composition for each seaweeds species.

Fatty acids	Chlorophyta								Phaeophyta									Rhodophyta					
	G1	G2	G3	G4	G5	G6	G7	G8	B1	B2	B3	B4	B5	B6	B7	B8	B9	R1	R2	R3	R4	R5	R6
C14:0	–	0.51	1.44	–	–	–	4.1	–	0.81	4.1	2.84	0.62	–	–	3.31	–	–	–	–	–	1.12	–	–
C16:0	–	0.31	3.71	1.44	1.57	–	10.62	9.91	15.11	0.61	10.26	1.24	38.52	1.55	0.61	17.23	9.51	22.63	9.95	–	14.55	20.21	8.53
C18:0	–	0.32	–	0.45	0.53	–	–	–	–	1.11	1.37	1.12	–	0.96	–	–	0.53	–	1.19	–	2.13	2.14	–
C20:0	–	–	–	0.36	–	–	–	–	–	–	–	–	–	0.27	0.88	–	–	–	–	–	–	–	–
∑ SFA	–	1.14	5.15	2.15	2.1	–	14.72	9.91	15.92	5.82	13.47	2.98	38.52	2.78	4.80	17.23	10.04	22.63	11.14	–	17.80	22.35	8.53
C16:1n7	1.01	1.31	0.76	2.84	2.49	1.04	3.65	1.58	3.52	2.43	1.46	1.21	–	1.75	16.31	1.69	2.3	–	–	–	–	–	0.22
C18:1n7	–	0.01	1.16	0.03	0.09	–	–	–	0.26	1	0.34	–	–	0.22	0.67	0.06	0.06	0.38	–	–	0.56	0.55	0.79
C18:1n9	0.56	–	0.13	–	–	0.53	–	–	–	–	–	–	1.39	0.03	–	0.15	0.13	–	–	–	0.11	0.15	–
C20:1n9	0.29	6.22	2.63	4.24	9.72	0.34	–	0.81	0.17	0.15	0.22	–	–	0.09	0.37	–	–	–	1.36	–	0.33	–	0.26
∑ MUFA	1.76	7.54	4.68	7.11	12.3	1.91	3.65	2.39	3.95	3.58	2.02	1.21	1.39	2.09	17.35	1.9	2.49	0.38	1.36	–	1	0.7	1.27
C18:2n6	8.41	8.13	7.04	8.23	7.68	8.61	8.69	8.68	6.03	6.42	6.18	7.09	3.45	6.68	6.48	7.25	6.35	8.51	8.65	9.11	7.37	8.03	8.35
C18:3n6	3.18	2.33	5.35	2.18	3.45	3.29	1.88	1.84	1.78	2.21	2.41	1.2	5.91	1.71	1.63	1.83	1.75	0.59	1.98	1.52	2.02	1.84	1.98
C20:3n6	2.37	0.02	3.22	0.07	1.86	2.77	–	2.37	21.21	0.08	11.25	17.47	0.35	14.95	7.7	0.07	13.66	3.38	2.86	6.68	0.07	4.91	6.92
C20:4n6	–	0.45	0.3	0.14	–	–	–	–	0.32	0.72	0.26	–	–	0.16	0.22	0.15	0.54	–	0.17	–	0.37	0.24	–
C22:5n6	–	0.03	–	0.03	–	–	–	–	–	–	0.07	–	–	–	–	–	–	–	–	0.67	–	–	–
C18:3n3	–	0.05	–	–	–	–	–	–	–	0.05	–	–	–	0.04	–	–	–	–	–	–	–	–	–
C20:5n3	0.83	0.91	0.76	0.83	0.96	0.9	–	0.34	1.92	0.51	1.25	0.72	0.58	1.05	–	1.56	0.64	0.25	–	–	0.34	0.63	0.84
C22:5n3	–	–	–	–	–	–	–	–	–	–	–	–	–	0.05	–	–	–	–	–	–	–	–	–
C22:6n3	0.15	–	0.36	0.29	–	0.17	–	–	1.07	0.24	1.06	0.53	5.87	0.82	–	–	0.67	–	–	–	–	–	–
∑ PUFA	14.67	11.92	17.03	11.77	13.95	15.74	10.84	13.23	32.33	10.23	22.48	27.01	16.16	25.46	16.03	10.86	23.61	12.73	13.66	17.98	10.17	15.65	18.10
n6-PUFA	13.69	10.96	15.91	10.65	12.99	14.67	10.84	12.89	29.34	9.43	20.17	25.76	9.71	23.50	16.03	9.30	22.30	12.48	13.66	17.98	9.83	15.02	17.25
n3-PUFA	0.98	0.96	1.12	1.12	0.96	1.07	–	0.34	2.99	0.8	2.31	1.25	6.45	1.96	–	1.56	1.31	0.25	–	–	0.34	0.63	0.84

(G1) *Ulva* *lactuca*, (G2) *Caulerpa* *racemosa,* (G3) *Halimeda* *opuntin*, (G4) *Caulerpa* *racemosa*, (G5) *Caulerpa* *sertularioides*, (G6) *Ulva* *lactuca*, (G7) *Chaetomorpha* *antennina*, (G8) *Chaetomorpha* *crassa*, (B1) *Padina* *antillarum*, (B2) *Sargassum* *illicifolium*, (B3) *Sargassum* *polycystem*, (B4) *Turbinaria* *ornata*, (B5) *Stoechospermum* *polypodioides*, (B6) *Sargassum* *illicifolium*, (B7) *Sargassum* *illicifolium*, (B8) *Padina* *antillarum*, (B9) *Sargassum* *illicifolium,* (R1) *Gracilaria* *corticata,* (R2) *Gracilaria* *corticata*, (R3) *Acanthophora* *spicifera*, (R4) *Gelidiopsis* *variabilis*, (R5) *Gracilaria* *corticata*, (R6) *Jania* *adhaereus.*

**Table 5:** Fatty composition values and the respective Green, brown and Red seaweed group (mean ± SE) %.

Fatty acids		Chlorophyta	Phaeophyta	Rhodophyta
Myristic	C14:0	0.70 ± 0.50	1.29 ± 0.55	0.18 ± 0.28
Palmitic	C16:0	3.44 ± 1.58	10.51 ± 4.18	12.64 ± 3.64
Stearic	C18:0	0.43 ± 0.10	0.56 ± 0.26	0.91 ± 0.48
Arachidic	C20:0	0.06 ± 0.03	0.12 ± 0.11	–
	∑ SFA	4.63 ± 0.55	12.48 ± 1.22	13.73 ± 1.46
Palmitoleic	C16:1n7	1.71 ± 0.42	3.41 ± 1.64	0.04 ± 0.04
Vaccenic	C18:1n7	0.16 ± 0.14	0.29 ± 0.11	0.38 ± 0.13
Oleic	C18:1n9	0.08 ± 0.07	0.19 ± 0.15	0.04 ± 0.03
Eicosenoic	C20:1n9	2.99 ± 1.25	0.11 ± 0.04	0.32 ± 0.21
	∑ MUFA	4.95 ± 1.38	3.99 ± 1.70	0.78 ± 0.22
**Linoleic**	C18:2n6	8.18 ± 1.04	6.21 ± 0.37	8.33 ± 0.24
γ-Linolenic	C18:3n6	2.54 ± 0.55	2.27 ± 0.47	1.65 ± 0.22
Homo-γ-linolenic	C20:3n6	1.28 ± 0.50	9.64 ± 2.67	4.14 ± 1.06
Arachidonic	C20:4n6	0.11 ± 0.06	0.26 ± 0.08	0.13 ± 0.06
Dpan-6	C22:5n6	0.01 ± 0.005	0.01 ± 0.01	0.11 ± 0.11
α-Linolenic	C18:3n3	0.01 ± 0.01	0.01 ± 0.01	–
EPA	C20:5n3	0.59 ± 0.14	0.91 ± 0.20	0.34 ± 0.14
Dpan-3	C22:5n3	–	0.005 ± 0.005	–
DHA	C22:6n3	0.10 ± 0.05	1.14 ± 0.61	–
	∑ PUFA	11.78 ± 1.96	20.53 ± 0.43	14.71 ± 0.31
	n6-PUFA	11.08 ± 1.09	18.46 ± 0.47	14.37 ± 0.32
	n3-PUFA	0.70 ± 0.18	02.07 ± 0.62	0.34 ± 0.14

– ; not available.

Total saturated fatty acids (SFAs) = the sum of C8 to C20.

Total mono unsaturated fatty acids (MUFAs) = the amount of C18:1.

Total poly unsaturated fatty acids (PUFAs) = the sum of C18:2 and C18:3.

Data are expressed as values: mean ± SE and analysed by one-way analysis of variance.

Sri Lankan seaweeds are largely unknown in terms of their elemental composition. We have identified 17 elements/minerals present in the 15 Sri Lankan seaweeds. The mineral concentrations are listed in Table 7. Due to the inherent difficulty in predicting where minerals are accumulated, the data discussed here are primarily from a human consumption perspective. The levels of these elements in seaweed have been linked to both risks and potential health benefits. Our study found that cadmium content did not exceed in food supplements exclusively containing dried seaweed. The highest concentrations of Cu, Zn, Co, Mo, Mg were recorded in *Ulva* *lactuca* (Table 6). Considerable concentrations of K and Ca were also observed in all the groups of seaweeds (Table 7).

**Table 6:** The percentage as a mean value (mg/100 g) of mineral elements composition for each seaweeds species.

	Cu	Pd	Cd	Cr	K	Zn	Mn	Ni	Co	Fe	Mo	Li	Se	Na	Ca	Mg	Sr
**Chlorophyta**																	
G1	5.37	0.17	0.13	0.84	133.67	97.25	3.54	2.83	0.79	119.77	32.73	9.19	0.38	294.61	184.00	107.92	29.98
G2	0.05	0.19	0.04	0.72	119.25	4.40	0.21	2.04	0.08	35.70	2.60	0.05	0.55	20.48	190.33	68.77	4.81
G3	0.26	0.22	0.04	0.31	119.66	6.94	1.74	1.40	0.02	133.42	0.72	0.05	0.71	26.92	212.80	75.12	438.41
G4	0.21	0.18	0.54	0.76	123.83	14.23	2.01	0.76	0.06	172.47	0.77	0.43	0.76	55.98	204.99	66.11	29.58
G5	0.88	0.44	0.14	0.77	121.68	7.72	1.97	3.08	0.03	147.78	0.63	0.03	0.68	8.80	189.97	57.64	68.34
G6	0.10	0.26	0.02	0.44	124.72	5.96	0.23	0.86	0.01	41.72	1.34	0.10	0.47	71.82	172.00	65.51	2.99
G7	0.03	0.22	0.96	0.09	122.91	8.65	5.12	1.85	0.07	127.57	0.46	0.51	0.57	73.00	176.37	66.06	3.25
G8	0.25	0.16	0.01	0.09	127.94	14.92	1.88	2.14	0.00	60.69	2.36	1.18	0.58	304.45	183.82	77.72	5.59
**Phaeophyta**																	
B1	0.32	0.21	0.26	1.03	129.02	3.86	1.85	0.13	0.05	137.97	1.18	0.26	0.62	38.98	197.23	83.25	148.52
B2	0.41	0.14	0.02	0.18	127.47	4.58	0.58	2.76	0.02	48.50	1.13	1.26	0.49	64.55	198.15	84.73	55.57
B3	0.18	0.44	0.04	0.70	127.66	5.76	2.22	1.36	0.07	128.46	0.93	2.43	0.67	80.87	187.43	86.38	90.69
B4	0.75	0.25	0.03	0.84	123.20	6.17	1.33	0.95	0.01	73.95	0.74	1.88	0.56	78.94	179.45	84.08	250.84
B5	0.06	0.17	0.02	0.33	122.84	4.93	1.19	0.53	0.01	70.03	0.79	1.23	0.73	7.28	181.48	53.60	64.97
B6	0.60	0.35	0.05	0.07	119.07	4.69	1.23	0.19	0.01	46.67	0.49	1.06	0.58	64.22	181.66	80.14	27.02
B7	0.26	0.16	0.55	0.23	126.61	6.54	6.59	2.05	0.06	75.56	0.59	3.00	0.69	75.16	191.98	84.14	126.51
B8	0.06	0.18	0.04	0.42	129.81	3.30	2.16	1.58	0.03	157.53	0.47	1.00	0.72	64.54	202.16	64.48	41.80
B9	0.57	0.20	0.04	0.09	124.75	5.79	1.42	2.02	0.06	60.00	0.63	3.16	0.60	121.47	178.96	81.51	261.28
**Rhodophyta**																	
R1	0.18	0.15	0.02	0.52	124.92	3.73	0.26	2.41	0.04	38.05	1.78	2.32	0.48	126.75	178.84	73.12	3.80
R2	0.29	0.35	0.05	0.43	130.13	3.95	1.24	0.38	0.01	36.47	0.61	1.13	0.43	45.05	179.28	58.76	3.42
R3	0.57	0.17	0.03	0.06	124.80	5.53	2.60	0.86	0.03	55.99	0.62	0.33	0.49	43.46	177.11	57.73	3.08
R4	0.21	0.36	0.01	0.78	132.81	5.12	0.77	0.17	0.01	31.40	0.75	1.14	0.53	394.81	183.01	78.02	5.21
R5	0.28	0.23	1.65	0.08	125.82	8.61	1.12	1.15	0.01	49.48	0.95	1.93	0.73	67.06	176.05	58.54	7.45
R6	0.37	0.22	0.01	0.09	121.61	70.94	3.20	2.16	0.28	73.97	0.66	3.61	0.57	86.23	181.64	60.02	4.36

(G1) *Ulva* *lactuca*, (G2) *Caulerpa* *racemosa,* (G3) *Halimeda* *opuntin*, (G4) *Caulerpa* *racemosa*, (G5) *Caulerpa* *sertularioides*, (G6) *Ulva* *lactuca*, (G7) *Chaetomorpha* *antennina*, (G8) *Chaetomorpha* *crassa*, (B1) *Padina* *antillarum*, (B2) *Sargassum* *illicifolium*, (B3) *Sargassum* *polycystem*, (B4) *Turbinaria* *ornata*, (B5) *Stoechospermum* *polypodioides*, (B6) *Sargassum* *illicifolium*, (B7) *Sargassum* *illicifolium*, (B8) *Padina* *antillarum*, (B9) *Sargassum* *illicifolium,* (R1) *Gracilaria* *corticata,* (R2) *Gracilaria* *corticata*, (R3) *Acanthophora* *spicifera*, (R4) *Gelidiopsis* *variabilis*, (R5) *Gracilaria* *corticata*, (R6) *Jania* *adhaereus.*

**Table 7:** The percentage as a mean value and the respective green, brown and red seaweed group (mean ± SE) %.

Minerals	Chlorophyta	Phaeophyta	Rhodophyta
Cu (µg/L)	8.95 ± 6.46	3.56 ± 0.82	3.16 ± 0.57
Pd (µg/L)	2.29 ± 0.33	2.34 ± 0.34	2.47 ± 0.36
Cd (µg/L)	2.35 ± 1.20	1.18 ± 0.59	2.95 ± 2.78
Cr (µg/L)	5.02 ± 1.10	4.32 ± 1.15	3.29 ± 1.21
K (µg/L)	1254 ± 16.76	1256 ± 11.48	1267 ± 16.60
Zn (µg/L)	200.10 ± 111.10	50.69 ± 3.61	163.10 ± 109.50
Mn (µg/L)	20.87 ± 5.74	20.63 ± 5.91	15.32 ± 4.61
Ni (µg/L)	18.69 ± 2.97	12.86 ± 3.03	11.88 ± 3.75
Co (µg/L)	1.33 ± 0.94	0.34 ± 0.08	0.66 ± 0.44
Fe (µg/L)	1049 ± 182.7	887.40 ± 137.90	475.60 ± 64.47
Mo (µg/L)	52.02 ± 39.43	7.70 ± 0. 87	8.93 ± 1.84
Li (µg/L)	14.43 ± 11.15	16.95 ± 3.28	17.43 ± 4.68
Se (µg/L)	5.87 ± 0.45	6.29 ± 0.27	5.39 ± 0.43
Na (µg/L)	1070 ± 428.30	662.20 ± 103.70	1272 ± 549.80
Ca (µg/L)	1893 ± 48.63	1887 ± 29.76	1793 ± 10.77
Mg (µg/L)	731.10 ± 54.31	780.30 ± 37.50	643.70 ± 36.12
Sr (µg/L)	728.70 ± 528.20	1186 ± 290.40	45.52 ± 6.54

Data are expressed as values: mean ± SE of three replicates and analysed by one-way analysis of variance.

**Figure 2:**
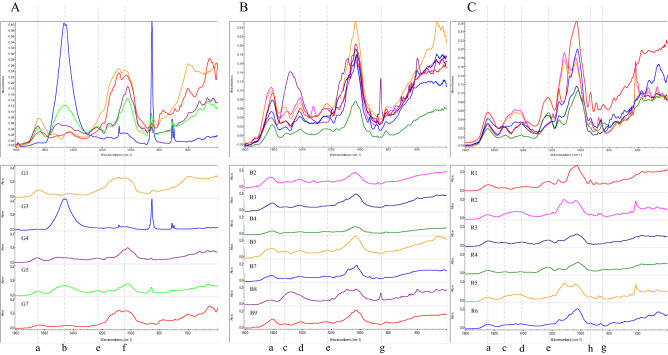
FT-IR spectra of raw seaweeds samples: (**A**) Chlorophyta, (**B**) Phaeophyta, (**C**) Rhodophyta. (**a**) C=C stretching (1650–1600 cm^−1^), (**b**) C–H bending; (1465–1450 cm^−1^), (**c**) N–O stretching (1550–1500 cm^−1^), (**d**) O–H bending; carboxylic acid (1440–1395 cm^−1^), (**e**) C–O stretching (1275–1200 cm^−1^), (**f)** S=O stretching (1070–1030 cm^−1^), (**g**) anomeric region (950–700 cm^−1^), (**h**) C–O–C bridge in 3,6-anhydro-l-galactose (930 cm^−1^). (G1) *Ulva* *lactuca*, (G2) *Caulerpa* *racemosa,* (G3) *Halimeda* *opuntin*, (G4) *Caulerpa* *racemosa*, (G5) *Caulerpa* *sertularioides*, (G6) *Ulva* *lactuca*, (G7) *Chaetomorpha* *antennina*, (G8) *Chaetomorpha* *crassa*, (B1) *Padina* *antillarum*, (B2) *Sargassum* *illicifolium*, (B3) *Sargassum* *polycystem*, (B4) *Turbinaria* *ornata*, (B5) *Stoechospermum* *polypodioides*, (B6) *Sargassum* *illicifolium*, (B7) *Sargassum* *illicifolium*, (B8) *Padina* *antillarum*, (B9) *Sargassum* *illicifolium,* (R1) *Gracilaria* *corticata,* (R2) *Gracilaria* *corticata*, (R3) *Acanthophora* *spicifera*, (R4) *Gelidiopsis* *variabilis*, (R5) *Gracilaria* *corticata*, (R6) *Jania* *adhaereus*.

The FTIR spectra of the seaweed species of Chlorophyta revealed medium absorption bands at 1633–1650 cm^−1^ (C=C stretching) and 1423–1504 cm^−1^ (O–H bending). The absorption peaks 1234–1238 cm^−1^, 1033–1142 cm^−1^, 856–844 cm^−1^, 598–776 cm^−1^ and 425–519 cm^−1^ indicated the existence of C–O stretching band (Fig. 2A), which were similar to the spectra reported previously^29^. The spectra of *Caulerpa* *racemosa* showed strong C=O stretching (δ-lactam) and N–O stretching (nitro compound) at 1650 cm^−1^ and 1504 cm^−1^, respectively. The absorption bands of Ocrophyta/Pheophyceae species were observed at 1607–1621, 1484–1519, 1417, 1323, 1223–1248, 932, 816–883, 712, and 464–599 cm^−1^ (Fig. 2B). Further, *Stoechospermum* *polypodioides* (B5), *Sargassum* *ilicifolium* (B7) species showed the strong IR absorption bands at 1620–1610 cm^−1^, which may be due to C=C stretching in α, β-unsaturated ketone. The bands at 1506 and 1519 cm^−1^ (strong) presented in *Sargassum* *ilicifolium* (B2) and *Stoechospermum* *polypodioides* (B5) were assigned to the N–O stretching. Bands in the region 1648–1638 cm^−1^ corresponded to the C=C stretching strong vibration of the red seaweeds, whereas strong peak at 1550–1500 cm^−1^ was assigned to the N–O stretching band related to the two red seaweed species, namely *Acanthophora* *spicifera* (R3) and *Ceratodictyon* *variabile* (formerly *Gelidiopsis* *variabilis*) (R4). The absorption peaks 930, 890 and 874–772 cm^−1^ revealed the existence of aromatic C-H bending band in *Gracilaria* *corticata* (R2 and R5) (Fig. 2C). However, C–Cl stretching was not found in the spectra of *G.* *corticata* (R1), which was collected from Ahangama costal area (5°58′40″ N, 80°22′28″ E).

## Discussion

Our study examined the chemical composition of 15 seaweed species in terms of their possible applications in food and health products. Many useful seaweed species have insufficient scientific data especially in terms of nutritional and medicinal benefits, despite their widespread use since the prehistoric times. Furthermore, several seaweed species may be found on the coasts of Asian countries and these seaweeds have been consumed since ancient times due to their high nutritional fibre and slow digesting carbs^30^. Currently, human consumption of green algae is (5%), brown algae is (66.5%) and red algae is (33%)^31^. The nutritional compositions of 15 seaweed samples tested in the present study belong to the green seaweed family Ulvaceae, Cladophoraceae, Caulerpaceae, and Halimedaceae. Brown seaweeds belong to the family Dictyotaceae, Sargassaceae. Red seaweeds belong to the family Gracilariaceae, Lomentariaceae, Corallinaceae, and Rhodomelaceae. The highest amount of crude protein was detected in the brown seaweeds,*Sargassum* *ilicifolium* (B9) when compared to green seaweeds,*Ulva* *lactuca* (G1) and *Caulerpa* *racemosa* (G2). Minimum protein content was recorded in brown seaweed species namely: *Stoechospermum* *polypodioides* (B5). According to the previous study, Ochrophyta/Phaeophyceae seaweed species contained higher protein content than the Rhodophyta and Chlorophyta seaweed species. The lowest amount of protein content was recorded in red algae: *Gracilaria* *corticata*^32^. It is important to note that the protein content of macroalgae is affected by both geographical location and seaweed species.

Present study revealed that the green alga: *Halimeda* *opuntia* (G3) yields highest ash content and *Ulva* *lactuca* (G1) contain the lowest amount of ash. High amount of ash was also observed in green seaweeds: *Chaetomorpha* *antennina* (G7), and brown seaweed: *Padina* *antillarum* (B8). A previous study showed that the ash content of edible red seaweeds ranged from 19.07 ± 0.61% to 34.00 ± 0.11%^33^. The intake of seaweed ash has been reported to prevent many diseases such as arthritis, fever, gout, fluid retention, bladder problems and constipation, and to improve intelligence^34^. Moreover, ash is used in treatment for childhood constipation^35^ and helps in maintaining bowel health^36^.

We found that green seaweeds species are rich sources of total dietary fiber: *Ulva* *lactuca* (G1) 81.59 ± 0.40%, and *Caulerpa* *racemosa* (G2) 81.70 ± 0.91%. The highest proportion of dietary fiber content has been reported in green seaweed, *Caulerpa* *chemnitzia*, which is significantly prominent when compared to dietary fiber levels of *Acanthophora* *spicifera* (Rhodophyta)*,* *Ulva* *intestinalis,* *Ulva* *rigida* (Chlorophyta), and *Sargassum* *wightii* (Ochrophyta/Phaeophyceae)^37^. In a study conducted by Lahaye, seaweed contained between 33 and 50% of dietary fiber^38^. Accordingly, the fiber content of green seaweed species is higher than those found in red and brown seaweeds. The consumption of this dietary fiber has been linked to the growth and protection of the beneficial intestinal flora^39,40^. Furthermore, the intake of dietary fiber helps in regulating calories and free cholesterol concentration, and reduces the risk of weight gain and obesity^41,42^.

Seaweeds are rich in PUFA from n3-PUFAs and n6-PUFAs series, thus, they could be an alternative valuable source of these compounds for human and animal health^43,44^. In this study we examined fatty acids profile by using the standard mix (Supplementary file 1). In general, a higher amount of PUFAs were reported from seaweeds compared to other groups of fatty acids including monounsaturated fatty acids (MUFAs). This study that omega-3 polyunsaturated fatty acids was not present in some red algae species. Long-chain PUFA, fatty acid C20:5n3 and C22:6n3 were found in seaweeds. Some PUFA are interesting since they serve as the precursors for the biosynthesis of regulating/signalling molecules like prostaglandins, thromboxanes and other bioregulators of many cellular processes^44^. However, there is significant difference (p < 0.05) between brown and red seaweeds in terms of SAFA, MUFA and PUFA. Amounts of saturated fatty acids (SAFAs) and PUFAs were higher in red seaweeds. MUFAs were higher in green seaweeds (4.95 ± 1.38%). In contrast, green seaweeds, like *Ulva* *australis* (formerly *Ulva* *pertusa*) (Chlorophyta), are characterised by the presence of hexadecatetraenoic (16:4 (n-3)), oleic (C18:1) and palmitic acids (C16:0)^45^. SAFAs and MUFAs were generally low in green and red seaweeds. SAFAs were higher in *Stoechospermum* *polypodioides,* *a* *brown* *seaweed* (B5). MUFAs was higher in *Sargassum* *ilicifolium* (B7), and PUFAs was detected highest in red alga *Acanthophora* *spicifera*, and green alga *Ulva* *lactuca*. The 20:4 n-6 and 20:5 n-3 fatty acids were the predominant PUFA found in red algae^46^ and hexadecatetraenoic acid is prominent in *Ulva* sp.^47^,^33^. *Acanthophora* *spicifera* (R3) contained 17.98% of PUFA followed by Linoleic, γ-linolenic, homo-γ-linolenic and docosapentaenoic acid (DPA).

Fatty acids such as docosahexaenoic acid (DHA) and eicosapentaenoic acid (EPA) are known to prevent cardiovascular diseases and inflammation caused by many chronic diseases^48^. Brown seaweeds contain higher levels of beneficial PUFAs, especially DHA and EPA. Furthermore, seaweeds contain a favorable ratio of omega-6 to omega-3 fatty acids. The red seaweeds contain 0.34 ± 0.14% n3-PUFAs and 14.37 ± 0.32% n6-PUFAs and the ratio of ω-3/ω-6 was 0.023%. Higher accumulation of n3-PUFAs was observed in brown seaweeds (02.07 ± 0.62%) and that of n6-PUFA was recorded in red seaweeds. The ω-3/ω-6 ratio is a good index for comparing the relative nutritional values of seaweed oils of different species, and a higher ratio of n-3/n-6 PUFAs has often been quoted as an index of higher nutritional value. Although many omega-3 fatty acids occur in nature, DHA and EPA are not synthesized by humans at a rate that can meet our metabolic needs, making a dietary source necessary^49^. Differences in fatty acids of marine seaweeds should only be considered with respect to species habitat. The difference in fatty acids composition varies depending upon the environmental conditions, especially water temperature^50^. Most of the seaweed varieties showed differences in their strain, appearance, geographical distribution and nutrient content.

Seaweeds are known as an excellent source of essential mineral, especially Na and Ca^38^. Minerals have a major role in synthesising hormones and enzymes^51^. Trace elements such as Iron (Fe), Copper (Cu), Cobalt (Co), Nickel (Ni), Zinc (Zn), Magnesium (Mg), Manganese (Mn), Molybdenum (Mo), Chromium (Cr), Lithium (Li), Selenium (Se), Fluorine (F) and Iodine (I) play an important role in healing and prevention of many diseases^52^. Sodium (Na), Calcium (Ca), Potassium (K) and Magnesium (Mg) are among the minerals which are present in significant amounts in marine algae^53^. Mineral concentrations such as Cu, Zn and Co were higher in the green alga,Mg and Sr were high in the brown alga,and Na was found highest in the red alga. Calcium is the leading mineral to help build strong bones and healthy teeth^54^. A higher level of Cd was found in red seaweeds when compared with other groups/classes of seaweeds. The presence of Cd could be due to exposure to human activities such as fishing and drainage system of the settlements in the coastal areas. This study explored the presence of heavy metals accumulation in red seaweeds. Heavy metals such as Lead (Pb), Arsenic (As), Cadmium (Cd) and Mercury (Hg) are toxic for health when exposed for a long time even at the lowest levels of their concentrations^55^. According to the World Health organization (WHO, 1989) report, the maximum allowed levels of heavy metals such as Pb, As and Cd in food and drug products are 10, 1.0 and 0.3 mg/kg, respectively^56^. Higher level of Pd (4.45 µg/L) has been shown in the brown algae, *Sargassum* *polycystum* (B3), and Cr was high in *Padina* *antillarum* (B1), species collected from south coast algae bed of Sri Lanka. The mineral fraction of some seaweeds has been shown up to 40% of dry matter, however mineral content of marine algae is recorded even higher than that of terrestrial plants^18^.

The FTIR spectroscopy is a fast, cost-effective, and extremely useful technique in identifying compounds present in the crude extracts^57,58^. Besides, FTIR spectra can be applied to distinguish agar and for estimating total sulphate content of carrageenans and agars^59,60^. A broad band at 3257–3333 cm^−1^ and medium signal at 2922–2928 cm^−1^ can be assigned to O–H, N–H functional groups and C–H stretching vibrations. Absorption peak ranging between the 4000—2000 cm^−1^ were common in all studied seaweeds and phycocolloid (carrageenans, agars and alginates). The absorption peaks at 3550–3200 cm^−1^ (Fig. 2) indicated the presence of O–H and the absorption peaks at 3000–2840 cm^−1^ can be attributed to C–H stretching vibrations (possibly −CH2 functional group), while 1650–1600 cm^−1^ were attributed to the C–H bending overtone band of aromatic compounds. Bands in the region 1440–1395 cm^−1^ corresponded to the asymmetric stretching vibration of the sulfate group O–H bending, whereas peak at 1275–1200 cm^−1^ was assigned to the C–O stretching band related to the C–O–SO_3_ group. The absorption peaks at 950–700 cm^−1^ revealed the existence of aromatic C–H bending band. One of the significant peaks was found at 930 cm^−1^ in the spectra of red seaweeds, which is associated with the stretching vibration mode of C–O–C bridge in 3,6-anhydro-l-galactose. IR absorption bands at 2960, 2920, 2845, 1640, 1370, 1250, 930, 900, 845, 805 and 705 cm^−1^ are used to obtain information on the structure of agars and carrageenans^61^. The seaweeds chemical composition identified by FT-IR and its vibrational spectra has allowed more accurate and an easier monitoring of chemical composition of seaweeds. This study outcomes can be applied in nutritional quality control and authentication of seaweeds.

The study shows that brown and green seaweeds are the most abundant seaweed species in the Sri Lankan coastal areas. Brown seaweed was a most common species in areas that were exposed to constant sea waves and where dissolved oxygen levels are high, whereas, the green seaweed species is the most common in deeper oceanic areas. Moreover, we observed that the most diverse seaweed communities are found close to the coast, where the depth gradient is changing their distribution patterns. The south location had a higher density of *Sargassum* *ilicifolum* species than other coastal locations. As well, a positive correlation (P < 0.05) was apparent between *Sargassum* spp. and other seaweeds^23^. Both north and south coastal areas exhibit marked differences in seaweed communities. For assertive correlations to be established between these locations' seaweed communities and their distributions, needs further studies.

There are large populations of *Sargassum* *illicifolium* in tropical and temperate oceans, a marine macroalgae of the Phaeophyceae family. It is a member of the marine genus Sargassaceae and order Fucales^8,62^. Recently, there has been an increasing discovery of seaweed metabolites presenting biological activities. For examples, it has been reported that their bioactive compounds possess antibacterial^63^, cytotoxic^64^, cell stimulation^8^, anticancer^65^ and immune-modulating properties^66^. *Sargassum* *illicifolium* has shown different amounts of protein when compared to other coastal species (B2, B6, B7, B7). Species of *Sargassum* spp (B6) collected from the north coastal area of Sri Lanka (Kankasanthurai, GPS point: N 09°48.592' E 080°02.546') contained the highest amount of protein. When compared with *Sargassum* *illicifolium* species collected from different coastal areas, *Sargassum* *illicifolium* (B7, collected from Negombo, GPS point: N 07°12.170' E 079°48.570') displayed the lowest amount of protein and lipid content.

As for red seaweeds, *Gracilaria* *corticata* (R1, R2) from the north and south areas have different ratio of ash, lipid, protein, and dietary fiber concentrations. Additionally, *Ulva* spp. showed different nutritional characteristics depending on the collection site. The results of this study indicated that nutritional properties vary depending on where they are collected. Due to their sensitivity to changes in water quality parameters, seaweed distribution patterns and abundance might change over time. Additionally, other environmental factors could influence the nutritional composition of seaweeds. A further study is highly recommended to explain how water quality parameters affect nutrition levels. According to our previous study, diluted *S.* *illicifolium* extracts induced promising cell proliferation and migration activity against the L929 cell line^23^. There were significant differences (p < 0.05) in cell proliferation activity between *S.* *illicifolium* samples collected from the south algae bed and samples collected from other algae beds of Sri Lanka. The results of the previous study indicated that the cytotoxic effects were also dose-dependent^67,68^. Furthermore, it was discovered that the nutritional value of seaweeds varied depending on geographical distribution^69,70^. Present investigation is the first report on the biochemical profile of seaweeds in Sri Lanka, and more research studies are needed in determining the nutritional profile and toxicological issues in using seaweed as a food and feed resource for humans and animals.

## Conclusions

This study is the first to highlight the nutritional significance of available seaweeds species in Sri Lanka and all 15 species were found to be sources of proteins, total dietary fiber, lipids and minerals. Seaweed species belonging to Ochrophyta/Phaeophyceae was the dominant marine algae that contained highest protein. Dietary fiber and ash contents were more prominent in seaweed species of Chlorophyta. The present investigation brings out comprehensive data on the biochemical (including fatty acids) and mineral compositions of three types of seaweeds. These baseline data will be beneficial in studying and establishing nutritional quality of seaweeds and their industrial applications. However, in vitro and in vivo therapeutic activities of most of these seaweeds and their compounds are yet to be determined. Thus, further studies would be required to determine specific phytocomponents and their health benefits.

## Supplementary Information

Supplementary Information.

## Data Availability

The datasets used and/or analysed in this study can be obtained from the corresponding author upon reasonable request.
