# Supplementary material for: Comparative analysis of proximate compositions, mineral and functional chemical groups of 15 different seaweed species
Source: Sci Rep. 2022 Nov 15;12:19610. doi: 10.1038/s41598-022-23609-8 (PMC9666456; doi:10.1038/s41598-022-23609-8)
Supplement: Supplementary file 1 — Supplementary Information. [file 41598_2022_23609_MOESM1_ESM.pdf]

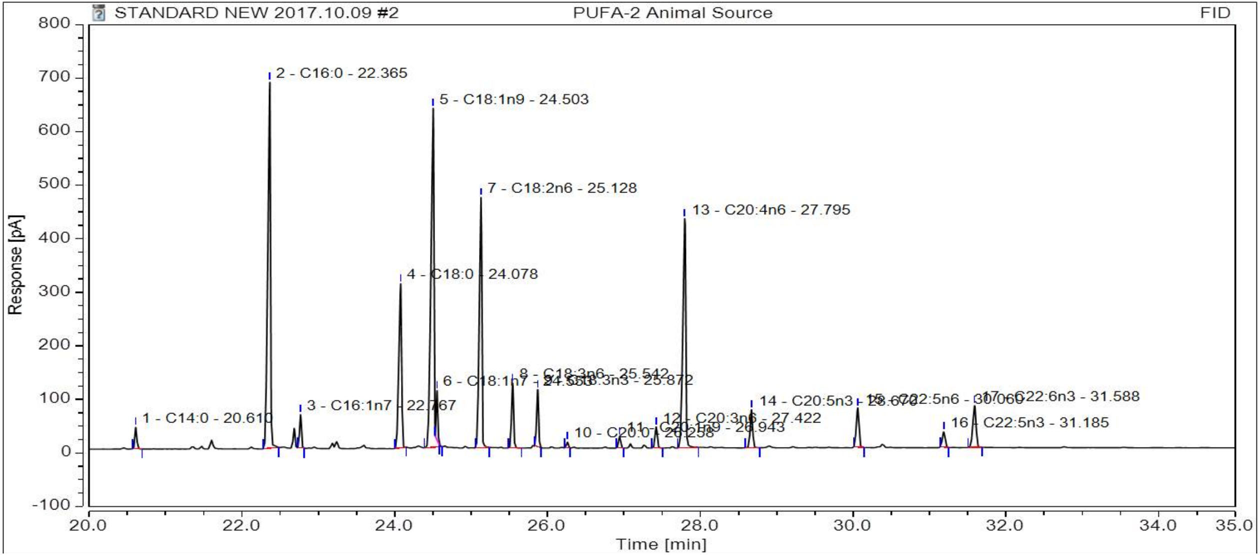

Figure X: A chromatogram of the fatty acid methyl esters (FAMEs) of the Stranded fatty acids Component FAME Mix (PUFA-2Animal source, catalog no: 47015-U). Main free fatty acid peaks at retention times: myristic acid 14:0; palmitic acid 16:0; palmitoleic acid C16:1n7; stearic acid 18:0; vaccenic acid C18:1n7; oleic acid 18:1n9; linoleic acid 18:2n6; γ -linolenic acid C18:3n6; α-linolenic acid C18:3n3; arachidic acid C20:0; eicosenoic acid C20:1n9; homo- γ –linolenic acid C20:3n6; arachidonic acid C20:4n6; dpan-6 acid C22:5n6; eicosapentaenoic acid (EPA) acid C20:5n3; dpan- 3 acid 22:5n3; docosahexaenoic acid (DHA) 22:6n3.
